# The Role of Multimodality Cardiovascular Imaging in Peripartum Cardiomyopathy

**DOI:** 10.3389/fcvm.2020.00004

**Published:** 2020-02-18

**Authors:** Fabrizio Ricci, Carlo De Innocentiis, Elvira Verrengia, Laura Ceriello, Cesare Mantini, Carla Pietrangelo, Flaviano Irsuti, Stefano Gabriele, Alberto D'Alleva, Mohammed Y. Khanji, Nay Aung, Giulia Renda, Matteo Cameli, Steffen E. Petersen, Ernesto Di Cesare, Sabina Gallina

**Affiliations:** ^1^Department of Neuroscience, Imaging and Clinical Sciences, “G. D'Annunzio” University, Chieti, Italy; ^2^Department of Clinical Sciences, Lund University, Clinical Research Center, Malmö, Sweden; ^3^Casa di Cura Villa Serena, Città Sant'Angelo, Pescara, Italy; ^4^Hypertension and Related Diseases Center, AOU-University of Sassari, Sassari, Italy; ^5^Cardiac Intensive Care and Interventional Cardiology Unit, Santo Spirito Hospital, Pescara, Italy; ^6^William Harvey Research Institute, NIHR Barts Biomedical Research Centre, Queen Mary University of London, London, United Kingdom; ^7^Barts Heart Centre, St. Bartholomew's Hospital, Barts Health NHS Trust, London, United Kingdom; ^8^Department of Life, Health and Environmental Science, University of L'Aquila, L'Aquila, Italy

**Keywords:** peripartum cardiomyopathy, cardiac magnetic resonance, pregnancy, heart failure, tissue characterization, echocardiography

## Abstract

The burden of pregnancy-related heart disease has dramatically increased over the last decades due to the increasing age at first pregnancy and higher prevalence of cardiovascular risk factors such as diabetes, hypertension, and obesity. Pregnancy is associated with physiological changes in the cardiovascular system, including hemodynamic, metabolic, and hormonal adaptations to meet the increased metabolic demands of the mother and fetus. It has been postulated that pregnancy may act as a cardiovascular stress test to identify women at high risk for heart disease, where the inability to adequately adapt to the physiologic stress of pregnancy may reveal the presence of genetic susceptibility to cardiovascular disease or accelerate the phenotypic expression of both inherited and acquired heart diseases, such as peripartum cardiomyopathy (PPCM). PPCM is a rare and incompletely understood clinical condition. Despite recent advances in the understanding of its pathogenesis, PPCM is not attributable to a well-defined pathological mechanism, and therefore, its diagnosis still relies on the exclusion of overlapping dilated phenotypes. Cardiac imaging plays a key role in any peripartum woman with signs and symptoms of heart failure in establishing the diagnosis, ruling out life-threatening complications, guiding therapy and conveying prognostic information. Echocardiography represents the first-line imaging technique, given its robust diagnostic yield and its favorable cost-effectiveness. Cardiovascular magnetic resonance is a biologically safe high-throughput modality that allows accurate morpho-functional assessment of the cardiovascular system in addition to the unique asset of myocardial tissue characterization as a pivotal piece of information in the pathophysiological puzzle of PPCM. In this review, we will highlight current evidence on the role of multimodality imaging in the differential diagnosis, prognostic assessment, and understanding of the pathophysiological basis of PPCM.

## Key Points

- Peripartum cardiomyopathy is a rare but potentially fatal disease requiring prompt identification and treatment.
- Cardiac imaging plays a pivotal role for the diagnosis, risk stratification, and follow-up of peripartum cardiomyopathy and related complications.
- Cardiovascular magnetic resonance is a high-throughput imaging modality providing relevant information for clinical decision-making and understanding of the pathophysiology underlying peripartum cardiomyopathy.

## Introduction

Cardiovascular diseases (CVDs) represent the main cause of maternal morbidity and mortality during or early after pregnancy in western countries (1–3). This remains an unacceptable price to pay for motherhood. After the initial description of heart failure (HF) development during pregnancy, the term “peripartum cardiomyopathy” (PPCM) was firstly introduced by Demakis et al. about 50 years ago (4). Since then, our knowledge of the pathophysiological framework of PPCM, although still incomplete, has noticeably increased, and substantial progress has been made toward improved diagnosis and treatment of this elusive disease.

According to the international guidelines (5–8), PPCM is defined by symptomatic left ventricular (LV) systolic dysfunction, with LV ejection fraction (LVEF) usually <45%, with or without LV enlargement, developing during the last month of pregnancy or in the first 5 months after delivery, abortion, or miscarriage in women without previously known heart disease. This definition entails two important requirements: firstly, the assessment of LV systolic dysfunction with cardiac imaging; secondly, the ascertainment of pre-existing maternal CVD.

PPCM is a rare disease with a generally accepted incidence of nearly 1 in 1,000–4,000 live births in western countries (9). However, the incidence is highly variable across different geographical areas (10), likely reflecting specific genetic susceptibility to different environmental influences. Currently, the incidence of PPCM is rising also in western countries, where it represents a non-negligible cause of pregnancy-associated HF and maternal death (11). At present, there is no recognized cause for PPCM, so that the diagnosis still relies on the exclusion of other specific conditions (5). Several hypotheses have been discussed (autoimmune, myocarditis, malnutrition, genetic altered prolactin formation), with familiar forms having been reported. Recently, a vasculo-hormonal hypothesis has been proposed where multiple signaling pathways may be deregulated in late gestation, causing angiogenic imbalance eventually leading to cardiac dysfunction in genetically predisposed individuals (10, 12–14). According to this hypothesis, the prolactin inhibitor bromocriptine shows promise in the treatment of PPCM (15); however, despite early promising results, specific biomarkers and therapeutic targets are lacking (16, 17).

**Figure 1:**
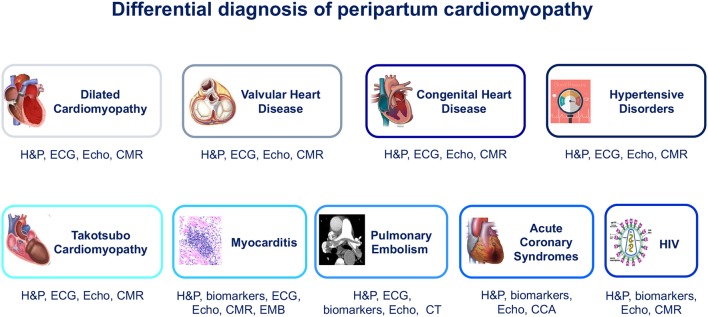
Differential diagnosis of peripartum cardiomyopathy. CCA, conventional coronary angiography; CMR, cardiac magnetic resonance; CT, computed tomography; ECG, electrocardiogram; EMB, endomyocardial biopsy; H&P, history and physical examination; HIV, human immunodeficiency virus; VHD, valvular heart disease.

**Figure 2:**
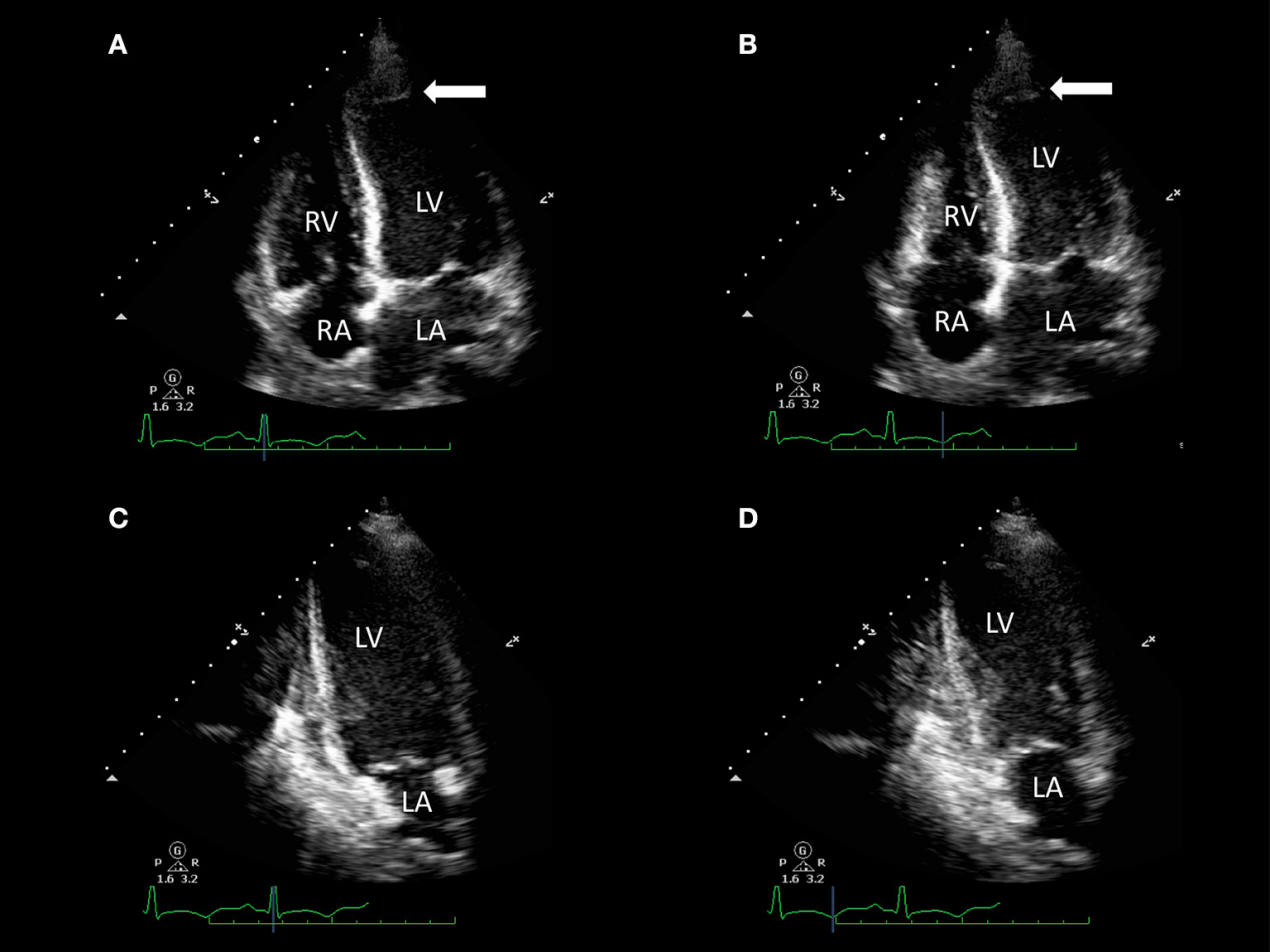
Transthoracic echocardiography of a 32-year-old woman with acute dyspnea 1 day after delivery. Apical four-chamber view [end-diastolic **(A)** and end-systolic **(B)** frames] and two-chamber view [end-diastolic **(C)** and end-systolic **(D)** frames] show akinesia of apical segments of the left ventricle (LV) and mild-to-moderate LV systolic impairment [LV ejection fraction (LVEF) 45%] with evidence of apical LV thrombus (arrows). Resting right ventricle (RV) function was preserved (fractional area change 48%).

**Figure 3:**
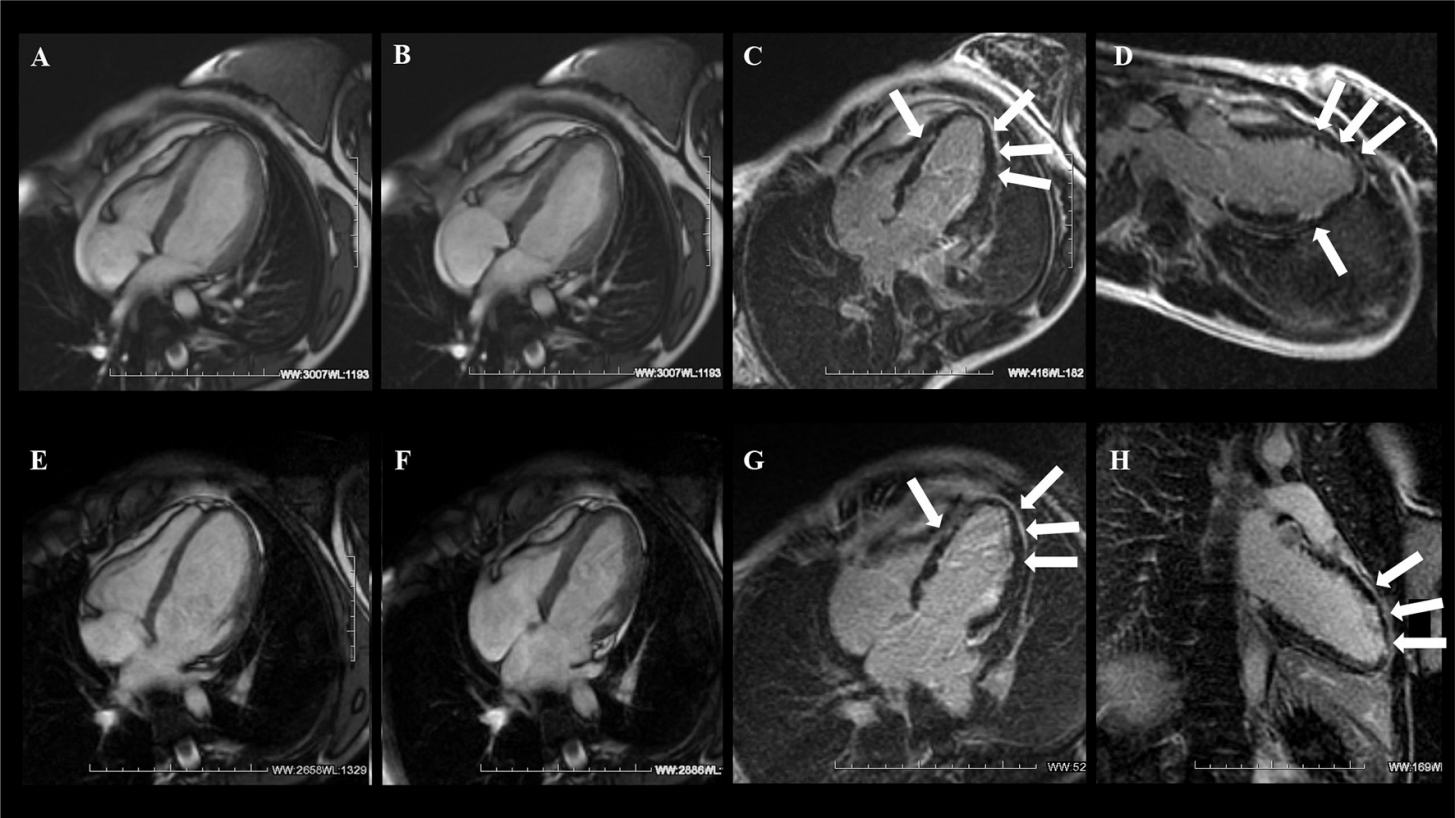
A 21-year-old woman with sudden onset of dyspnea 1 week after delivery and without prior medical history. CMR examination with cine balanced steady-state free precession (bSSFP) sequence four-chamber view [end-dyastole **(A)** and end-systole **(B)**] showed severely reduced LV systolic function without RV involvement. **(C,D)** T1-weighted inversion recovery gradient echo (T1w-IR-GRE) sequence acquired 10 min after gadolinium administration showed diffuse subendocardial late gadolinium enhancement (LGE) of the LV (arrows). At 6 months' follow-up, there was evidence of LV functional recovery **(E,F)** with diffuse LGE persistence **(G,H)**. Courtesy of Dr. Giovanni Donato Aquaro, Fondazione Toscana Gabriele Monasterio, Pisa, Italy.

**Figure 4:**
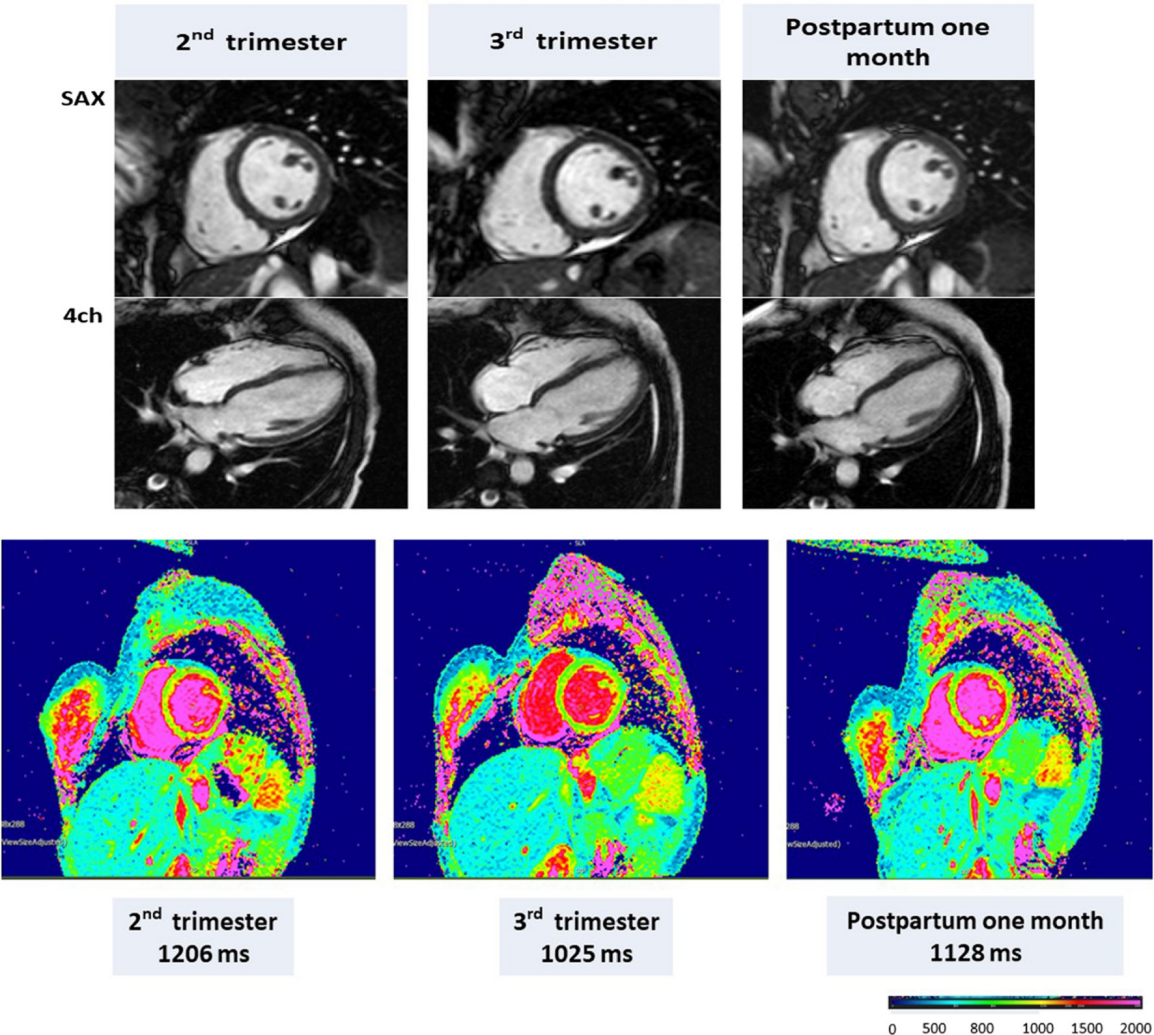
CMR in a representative pregnant woman with end-diastolic volume (EDV), end-systolic volume (ESV), ejection fraction (EF), and left ventricular mass (LVM), respectively, of 132 mL, 69 mL, 48.0%, and 106 g during the second trimester; 136 mL, 60 mL, 56%, and 95 g during the third trimester; and 112 mL, 57 mL, 54%, and 76 g at 1 month postpartum. Native T1 mapping (1.5 T) using MOLLI with a native T1 of 1,206 ms in the second trimester, 1,025 ms in the third trimester, and 1,128 ms at 1 month postpartum. Adapted from Nii et al. (21).

Along with patients' medical history, physical examination, electrocardiogram (ECG), and B-type natriuretic peptide assessment (18), cardiac imaging plays a key role for the clinical evaluation of peripartum women with symptoms and signs of HF (Figure 1, Table 1). Echocardiography is the first-line diagnostic imaging modality given its wide availability, biological safety, and robust diagnostic yield in HF patients (6, 19). Noticeably, comprehensive cardiopulmonary ultrasound examination allows biventricular systolic function assessment, early detection of subclinical hemodynamic derangements, monitoring of extravascular lung water and left atrial pressure, and prompt identification of complications such as thrombosis. Cardiovascular magnetic resonance (CMR) without administration of gadolinium-based contrast agents is a second-tier imaging modality that can be safely performed during pregnancy (20). CMR outperforms echocardiography (i) in the assessment of cardiac function, flow, and volumes, (ii) in the identification of intracardiac thrombi, and (iii) in detecting and monitoring signs of acute myocardial inflammation (Figures 2–**4**).

**Table 1:** Clinical workup of patients with suspected peripartum cardiomyopathy.

History and physical examination	Signs and symptoms of heart failure; blood pressure; exclusion of previous cardiovascular diseases; PPCM typically presents in the first 2 weeks after delivery
12-lead ECG	Atrial abnormalities; left ventricular hypertrophy; ST-T wave abnormalities; arrhythmias
Chest x-ray	Assessment of pulmonary congestion
Biomarkers	Natriuretic peptides and cardiac troponin usually elevated, but poor specificity; novel HF biomarkers (interest for research)
Echocardiography	LV systolic and diastolic dysfunction with or without LV dilatation; LV thrombosis; RV involvement; exclusion of pre-existing diseases (VHD, CHD); assessment of lung congestion
CMR	LV systolic and diastolic dysfunction with or without LV dilatation; intracardiac thrombosis; RV involvement; tissue characterization; differential diagnosis (myocarditis, Takotsubo, VHD, DCM)
Genetic testing	DCM and dystrophin mutations; X-linked disease; family counseling

*CHD, congenital heart disease; CMR, cardiac magnetic resonance; DCM, dilated cardiomyopathy; ECG, electrocardiogram; HF, heart failure; LV, left ventricle; RV, right ventricle; VHD, valvular heart disease; PPCM, peripartum cardiomyopathy*.

## Review Methodology

Authors performed a narrative review and searched Medline from January 1966 through August 2019 for potentially relevant articles using the following search terms: (peripartum cardiomyopathy OR pregnancy) AND (echocardiography OR cardiovascular magnetic resonance OR computed tomography OR nuclear OR cardiovascular imaging). References of selected papers were screened, searching for other potentially relevant publications. Websites, including escardio.org and ResearchGate, as well as reference lists of all identified articles, including hand-searching of reviews and previous meta-analyses, were also appraised for additional relevant studies. Four authors screened a list of potentially relevant articles based on title, abstract, and full-text review. A selection of the most relevant papers was finally included in the review according to authors' opinion.

## Echocardiography

Pregnancy produces prominent cardiovascular adaptations that become particularly evident in the last trimester and peripartum period. These changes include increase of LV mass, dilation of cardiac chambers, increase in cardiac output, and elevation of LV filling pressure due to fluid volume overload (6, 22). For its wide availability and low cost, echocardiography, also with recently introduced three-dimensional (3-D), tissue velocity imaging and myocardial strain analysis (23), is the most commonly used imaging method for cardiac chamber quantification (24). It provides real-time information on atrioventricular size and function, valvular apparatus, and other cardiac structures and forms the first-line imaging modality for the evaluation of pregnant women with suspected CVDs (6). Echocardiography plays a key role in the differential diagnosis between PPCM and other pregnancy-related cardiac diseases such as preeclampsia (25), in which LV systolic function is mostly unaffected, valvular heart diseases (VHDs), and congenital heart diseases (CHDs), and enables long-term monitoring of cardiac function (26) (Table 2).

**Table 2:** Value of cardiac imaging modalities in peripartum cardiomyopathy.

	**Echo**	**CMR**	**CT**	**Nuclear**
Differential between PPCM and pregnancy-related physiological cardiovascular adaptations	++	+++	–	–
Differential between PPCM and other cardiac conditions (DCM, Takotsubo, myocarditis, pregnancy-related MI)	++	+++	+(^**^)	++
Differential between PPCM and other extracardiac conditions (PE, amniotic fluid embolism)	++	+++	++(^***^)	–
Staging and monitoring disease progression	++	+++	–	–
Identification of intracardiac thrombosis	++	+++	++(^**^)	–
Risk stratification and prognostic evaluation	+++	+++(^*^)	+/–	–
Surveillance in subsequent pregnancies	+++	++	–	–
Assessment of specific disease pathways	–	++	–	–

* *Preliminary findings from small case series*.

** *Coronary CTA*.

*** *Pulmonary CTA*.

*CMR, cardiac magnetic resonance; CTA, computed tomography angiography; MI, myocardial infarction; PE, pulmonary embolism; PET, positron emission tomography; PPCM, peripartum cardiomyopathy; SPECT, single-photon emission computed tomography; CT, computed tomography*.

Although LV systolic dysfunction is observed in the vast majority of PPCM patients, as reduced LVEF is a major criterion for the diagnosis (5), several studies reported unexplained HF with preserved LVEF in peripartum women, suggesting the possibility of progressive myocardial disease development across different clinical stages (27–29) and raising the challenge for early diagnosis.

Strain has been validated as an important echocardiographic parameter with diagnostic and prognostic utility in various cardiac conditions. In this context, myocardial strain analysis would be the ideal tool for unmasking early myocardial impairment associated with various degrees of myocardial inflammation, oxidative stress, and fibrosis eventually driving the transition to overt HF. Accordingly, in a single-center experience, global longitudinal strain (GLS) was significantly lower in PPCM patients compared with healthy peripartum women regardless of LVEF values (28). This holds major promise in tracking early remodeling in PPCM and identifying a window of opportunity for preventive measures.

LV thrombosis is a common finding in PPCM, due to the coexistence of multiple predisposing factors. Although CMR has been proven to be superior to both transthoracic and transesophageal echocardiography in the detection of LV thrombi (30), echocardiographic evaluation of PPCM patients should always include a systematic assessment for this life-threatening complication. The use of 3-D techniques provides further morphological information compared to standard 2-D echocardiography and may be of additional benefit in the detection of intracardiac thrombi (31–33). The use of contrast echocardiography may improve the detection of thrombosis through the identification of an LV filling defect (34). Nevertheless, CMR remains more sensitive than echocardiography for the detection of intracardiac thrombus, especially in the case of mural or smaller thrombi (35, 36). Compared with late gadolinium enhancement (LGE) CMR as the reference standard, echocardiography has been shown to have a sensitivity of 24–37% when contrast is not used, with a higher sensitivity of <65% when contrast is used (36).

Despite PPCM-related outcome appearing to be favorable compared with other cardiomyopathies (37), with most patients showing full recovery within 6 months, current evidence for long-term outcome is based mostly on single-center studies or small registries (38). Echocardiography conveys important prognostic information useful for risk stratification (Table 3), and several echocardiographic parameters have been associated with worse outcomes or risk of relapse in subsequent pregnancies.

The results of the Investigations of Pregnancy-Associated Cardiomyopathy (IPAC) study, a prospective multicenter study on 100 PPCM patients, highlighted that baseline LVEF <30% and LV dilatation with a left ventricular end-diastolic diameter (LVEDD) >60 mm were strongly associated with lower rates of both 12-month event-free survival and LV functional recovery (39). Similar findings have been reproduced in other studies, although the effect of racial and ethnic differences requires future research (40–43).

PPCM is associated with risk of recurrence during subsequent pregnancies and requires adequate counseling (38). Several studies demonstrated that persistent LV systolic impairment is a strong predictor of poor cardiovascular outcome in this setting (44). In a retrospective study of 44 women undergoing a subsequent pregnancy, the risk of adverse maternal or fetal outcomes was substantially higher in those with persistent LV systolic dysfunction (45). Larger single-center and multicenter prospective studies (46, 47) confirmed the prognostic impact of LVEF on the outcome of subsequent pregnancies, where partial recovery of LV function (LVEF <50%) was a harbinger of higher mortality. Despite full recovery of resting LVEF, the residual risk of recurrent HF has been documented and linked to the persistence of subclinical systolic dysfunction. This has been shown in a cohort of 29 women with previous PPCM presenting with lower tissue Doppler velocities and GLS of both the LV and right ventricle (RV) despite full LVEF recovery 12 months after the index event (48).

The role of pharmacological stress testing during pregnancy is currently limited, with conflicting data on the prognostic value of LV contractile reserve assessment by dobutamine stress echocardiography (DSE). In a series of seven patients, the presence of inotropic contractile reserve—defined as the improvement of wall motion score in ≥2 segments—was a better predictor than resting LVEF of LV functional recovery during follow-up (49). Conversely, in another cohort of nine patients, resting LVEF was the sole predictor of LV functional recovery at follow-up (50). Small sample sizes, different DSE protocols, and clinical characteristics of the two patient populations may explain the conflicting results. Further larger prospective studies are needed to clarify the role of DSE for risk stratification and prognostic assessment of PPCM.

RV involvement identifies a PPCM phenotype with worse outcome. Echocardiography is a cost-effective alternative to CMR for RV function assessment in PPCM patients, particularly in those countries where PPCM is relatively more frequent and with limited economic resources (51). In the large IPAC cohort (52), the presence of RV systolic dysfunction was associated with persistent LV systolic impairment and poor outcome, and among different RV functional parameters, fractional area change (FAC) was the sole independent predictor of clinical outcome. The prognostic significance of baseline RV dysfunction was confirmed in a retrospective cohort study analysis of 53 patients (43) where moderate-to-severe RV systolic dysfunction was associated with 12-month higher risk of left ventricular assist device (LVAD) implantation, heart transplantation, or death. This evidence would suggest routine RV functional evaluation with FAC calculation in order to classify PPCM patients at higher risk of hemodynamic instability that may benefit from early advanced HF therapy. Bromocriptine treatment seems to be a promising option also in the context of biventricular dysfunction, although further placebo-controlled trials are needed (53).

Echocardiography is useful for counseling purposes and for risk stratification of PPCM women undergoing a subsequent pregnancy, individuating those that may benefit more from strict follow-up and timely treatment, also in the case of apparent full recovery of global LV systolic function (Table 2 and Figure 2).

## Cardiovascular Magnetic Resonance

In the last decade, CMR has emerged as the gold standard technique for cardiac chamber quantification and flow assessment, but the key advantage of CMR is its unique ability to provide information about the tissue composition by myocardial tissue characterization with high spatial resolution, excellent accuracy, and reproducibility (54). Accordingly, CMR is the best alternative imaging modality in patients with non-diagnostic or doubtful echo, but also an indispensable diagnostic tool in the workup of dilated cardiomyopathy (DCM). CMR has demonstrated its usefulness in the evaluation of physiological and pathological maternal cardiovascular adaptations to pregnancy (55), with reference values for ventricular volumes and tissue composition in healthy pregnancies and postpartum states having been published (21, 56). CMR imaging is considered safe during pregnancy, as magnetic energy has been shown not to be harmful for the developing fetus. Conversely, the effect of gadolinium-based contrast agent administration on the fetus is not well-established, so that its administration during pregnancy preferably should be avoided, especially during the first trimester (57, 58). Nevertheless, gadolinium-based contrast agents are considered safe during breastfeeding (59). LGE imaging allows identification of myocardial scarring (60). Despite the absence of a specific PPCM scar pattern, CMR may be useful for exclusion of other diseases such as myocarditis or Takotsubo syndrome that may have substantial phenotypic overlap with PPCM (61). Morpho-functional assessment of the RV is way more accurate by CMR and may be of prognostic value in PPCM. CMR therefore should be considered, in addition to ultrasound assessment when RV involvement is suspected, in the workup of alternative diagnosis—such as myocarditis or LV thrombosis—or in the case of poor-quality ultrasound images. However, regrettably, CMR remains underused for diagnosis and risk stratification of PPCM in clinical practice (Table 2).

The first case of CMR assessment of PPCM was described in 2008 (62). Authors reported the presence of LV systolic dysfunction with non-ischemic LGE and borderline myocarditis at endomyocardial biopsy, in keeping with the hypothesis of an inflammatory process playing in the background. CMR examination was performed at 2 and 10 months after symptom onset with evidence of significant LGE reduction at follow-up. Another study found that the presence of LGE was an independent predictor of persistently reduced LV systolic function at follow-up (63).

In another case series, CMR could identify neither LGE nor signs of myocardial edema in the entire study cohort. However, it should be acknowledged that CMR was performed beyond 2 weeks from HF onset in most patients, and nearly 40% of the study population presented with pregnancy-induced hypertension, hence not entirely meeting PPCM diagnostic criteria (64).

Supporting the hypothesis of the time dependence of detection of tissue composition alteration, both diffusely increased T2 and early gadolinium enhancement ratios (markers of myocardial edema) have been documented at the initial evaluation of women with PPCM (65). LGE was not present in the acute phase, but it has been reported during follow-up in a woman with persistently elevated T2 ratio. The presence of both LGE and high T2 ratio was also associated with persistently impaired LV function.

In a series of 10 patients with established PPCM, CMR revealed LGE with non-ischemic distribution in 40% of cases. LGE was associated with higher risk of HF hospitalization and HF exacerbation during subsequent pregnancies (66). An elevated T2 ratio was detected in one of the four patients that underwent examination in the acute phase, with complete resolution at 4 months and in the absence of residual LGE.

**Table 3:** Imaging predictors of poor outcome in peripartum cardiomyopathy.

Severe left ventricular systolic dysfunction
Right ventricular systolic dysfunction
Intracardiac thrombi
Left ventricular end-diastolic diameter >60 mm
Low tissue Doppler imaging velocities
Myocardial edema (increased T2 ratio, EGE ratio, T2 mapping)
Late gadolinium enhancement (presence, extent, and persistence)
Abnormal T1 mapping indices (?)
Microvascular disease (?)
Absence of contractile reserve by stress imaging (?)

In a larger CMR study enrolling 34 PPCM women, most patients presented with LVEF <35%, LV dilatation, and regional wall motion abnormalities (67). The observed prevalence of non-ischemic LGE and myocardial edema in this population was 70 and 25%, respectively, which was significantly higher compared with other non-ischemic cardiomyopathies. Interestingly, areas of pathological tissue composition matched regional wall motion abnormalities and were mainly located at the basal and mid-anteroseptal LV segments, in keeping with a reverse-Takotsubo pattern. About one-third of patients showed impaired RV systolic function, which was a harbinger of reduced chance of functional recovery. In another prospective multicenter study (60), the prevalence of LGE was quite low, likely because of the different time interval between symptom onset and CMR between studies.

CMR is also useful in the detection of intracardiac thrombosis. LV thrombosis is a serious complication of PPCM with potentially devastating consequences. The reported prevalence of LV thrombosis is highly variable, ranging between 10 and 30% (9). PPCM confers a higher risk of systemic thromboembolism compared to other cardiomyopathies (68) due to prolonged immobility, postpartum hypercoagulability, and reduced LV function. CMR is more accurate than transthoracic echocardiography for the identification of LV thrombus (30, 35), allowing early treatment and adequate follow-up. In a Danish study aimed at comparing the long-term CMR findings among PPCM, preeclampsia, and uncomplicated pregnancies (69), it has been shown that the prevalence of LGE was quite low and the rate of delayed functional recovery was noticeably high in PPCM, raising the issue of optimal timing for ICD implantation in primary prevention. Furthermore, CMR documented persistent diastolic dysfunction in PPCM compared with preeclampsia.

Current evidence suggests that the utility of phenotyping PPCM by CMR imaging is manifold and should be aimed at establishing a correct diagnosis, improving risk stratification, detecting thrombotic complications, and planning appropriate follow-up (Figures 3, 4). Furthermore, tissue characterization by CMR might be helpful to clarify the complex and multifactorial pathogenesis of PPCM distinguishing specific disease processes in PPCM and related phenocopies (70).

## Cardiac Imaging Modalities With Ionizing Radiation

Ionizing radiation exposure during pregnancy may be harmful for both mother and fetus. In pregnant or lactating women, the risk profile of radiation exposure is similar to that of non-pregnant women, with the exception of breast tissue. Given pregnancy-induced proliferation, it has been postulated that breast tissue of such patients is more sensitive to radiation, although the entity of this effect remains unclear (71). The consequences of fetal radiation exposure include spontaneous abortion, teratogenesis, and carcinogenesis. The effect depends on both gestational age and absorbed dose. While there is no minimum threshold below which there is no increased cancer risk, a fetal absorbed dose of <50 mGy is actually considered safe in terms of abortive and teratogen effects (72, 73). With the adoption of dose-reduction protocols, such a threshold is usually not exceeded in most radiographic procedures unless repeated examinations are necessary (74). Currently used iodinated contrast agents are able to cross the placenta, but no teratogenic effects have been reported (75). Such drugs should be used when they may contribute to diagnosis and benefits outweigh the risks (74).

Non-ionizing imaging methods should be preferred whenever possible, and if radiographic or nuclear techniques are necessary, the risk/benefit profile must be carefully evaluated on an individual basis without exposing patients to potentially life-threatening consequences of delayed diagnosis.

Chest x-ray represents the initial radiographic imaging method in the evaluation of the vast majority of pregnant women with HF when other radiation-free techniques are unable to clarify symptoms (6). Both maternal and fetal absorbed radiation doses are very low and without clinical consequence.

The role of other radiographic and nuclear imaging methods in PPCM is limited, and these techniques are usually not required. However, computed tomography (CT), ventilation/perfusion (V/Q), scan or invasive coronary angiography (ICA) may be necessary in order to establish a differential diagnosis between ppCM and other diseases with acute HF presentation, such as pulmonary embolism (PE), amniotic fluid embolism, and acute coronary syndromes (ACSs).

PE is a well-known complication of pregnancy-associated hemodynamic and metabolic changes and represents a relevant cause of maternal death (76, 77). It may manifest with acute dyspnea and in severe cases with cardiogenic shock. In the case of elevated clinical suspicion and negative lower-limb Doppler ultrasound results, CT-pulmonary angiography (CTPA) is usually recommended, with generally low fetal radiation dose. A perfusion lung scan leads to similar or even lower fetal radiation exposure and a decreased maternal breast absorbed dose and may therefore be preferred to CTPA especially in the presence of normal findings in chest x-ray examination, when the ventilation portion of the V/Q scan may be eliminated with similar diagnostic accuracy to CTPA (70, 78, 79). However, the best management of PE during pregnancy is still being debated (80). Non-contrast CMR using bright blood techniques such as balanced steady-state free precession sequences may be also considered as a viable alternative approach for ruling out PE in selected cases (81–83).

Although pregnancy is associated with an increased risk of ACS compared with age-matched non-pregnant women, ACSs are relatively uncommon during pregnancy (84). In addition to coronary atherosclerosis, other non-atherosclerotic etiologies, particularly spontaneous coronary artery dissection (SCAD), represent a relevant cause of ACS in peripartum patients according to age and risk factors (85, 86). Pregnancy-related ACS is most common during the third trimester, although clinical presentation is the same as in the non-pregnant population, and management in pregnancy is similar to that in the general population, including revascularization techniques (6).

In the setting of suspected chronic coronary syndrome related to obstructive coronary artery disease (CAD), the choice of the optimal non-invasive diagnostic test should be guided by pre-test probability (PTP) and patient characteristics (87). Coronary CT angiography (CTA) is the preferred initial test in patients with low clinical likelihood of CAD and may be considered also in pregnant patients as an alternative to ICA. Although pharmacological stress echocardiography and stress CMR (i.e., adenosine, regadenoson, or dypiridamole) would represent the functional test of choice in the vast majority of symptomatic patients with intermediate PTP of CAD, they are not recommended during pregnancy, as vasodilators may lead to maternal hypotension and placental hypoperfusion. After delivery, should a myocardial perfusion test be necessary, nuclear medicine imaging [single-photon emission computed tomography (SPECT)/positron emission tomography (PET)] can be considered, especially with reduced radiotracer doses. A temporary interruption of breastfeeding, depending on the half-lives of the radiotracers, is advisable whether or not performed during lactation (74). ICA may be required to establish a definite diagnosis and start appropriate treatment in the case of typical angina and/or evidence of myocardial ischemia.

## Gaps in Knowledge and Future Perspectives

A correct diagnosis of PPCM is key to start early treatment, plan appropriate follow-up, and provide adequate counseling. Recent data support the use of specific drug therapy with the prolactin inhibitor bromocriptine, although most studies are small-sized and with short-term follow-up. Future studies are needed to clarify the clinical importance and prognostic relevance of subclinical cardiac dysfunction at different stages of the disease by means of advanced echo modules including tissue Doppler imaging, speckle strain analysis, 3-D, contrast, and stress echocardiography. Further, 3-D echocardiography holds promise toward early detection of subclinical RV dysfunction and improved sensitivity for imaging of intracardiac thrombosis. Finally, the prognostic value of echocardiographic parameters other than LVEF or LV size requires further evaluation (Tables 2, 3).

Although LGE is a robust outcome predictor in several non-ischemic cardiomyopathies, available evidence for PPCM highlighted conflicting results on scar prevalence in the absence of any specific scar pattern. This might be secondary to the small number of subjects enrolled, the heterogeneity of study design, and the poorly defined pathophysiology underlying PPCM that may be different across specific patient subgroups. Advanced tissue characterization by native and post-contrast parametric mapping and MR fingerprinting may provide valuable information about specific disease process underlying PPCM and should be the objective of future investigations (Table 3). Given the relative rarity of PPCM, prospective multicenter registries would be helpful to identify novel prognostic markers in PPCM, including CMR markers of fibrosis, genetic markers, and biomarkers, and to clarify the role of multimodality cardiac imaging in the assessment of PPCM. In this way, novel and effective strategies for risk stratification will help counseling for future pregnancies, tailored decisions on HF prevention or treatment may be substantially improved, and potential targets for disease-modifying therapeutic intervention using existing or novel drug therapy may be eventually demonstrated.

## Author Contributions

FR: conceptualization, literature review, and writing—original draft. CD, EV, LC, CM, CP, FI, SGab, and AD'A: literature review and writing—original draft. MK, NA, MC, GR, ED, and SP: review and editing. SGal: conceptualization, project administration, and supervision.

### Conflict of Interest

The authors declare that the research was conducted in the absence of any commercial or financial relationships that could be construed as a potential conflict of interest.
